# Novel Insights into Concepts and Directionality of Maternal–Fetal Cholesterol Transfer across the Human Placenta

**DOI:** 10.3390/ijms19082334

**Published:** 2018-08-09

**Authors:** Sampada Kallol, Xiao Huang, Stefan Müller, Corneille Edgar Ontsouka, Christiane Albrecht

**Affiliations:** 1 Institute of Biochemistry and Molecular Medicine, Faculty of Medicine, University of Bern, CH-3012 Bern, Switzerland; sampada.kallol@ibmm.unibe.ch (S.K.); xiao.huang@ibmm.unibe.ch (X.H.); corneille.ontsouka@ibmm.unibe.ch (C.E.O.); 2 Swiss National Center of Competence in Research, NCCR TransCure, University of Bern, CH-3012 Bern, Switzerland; 3 Department of BioMedical Research, University of Bern, CH-3012 Bern, Switzerland; stefan.mueller@dbmr.unibe.ch

**Keywords:** ABC transporter, cholesterol, fetal development, pregnancy, trans-placental transport, trophoblasts

## Abstract

Cholesterol is indispensable for cellular membrane composition and function. It is also a precursor for the synthesis of steroid hormones, which promote, among others, the maturation of fetal organs. A role of the ATP-binding-cassette-transporter-A1 (ABCA1) in the transport of maternal cholesterol to the fetus was suggested by transferring cholesterol to apolipoprotein-A-1 (apo-A1), but the directionality of the apoA-1/ABCA1-dependent cholesterol transport remains unclear. We isolated primary trophoblasts from term placentae to test the hypotheses that (1) apoA-1/ABCA1 dispatches cholesterol mainly towards the fetus to support fetal developmental maturation at term, and (2) differentiated syncytiotrophoblasts (STB) exert higher cholesterol transport activity than undifferentiated cytotrophoblasts (CTB). As experimental models, we used (1) trophoblast monolayers grown on Transwell^®^ system consisting of apical (maternal-like) and basal (fetal-like) compartments, and (2) trophoblasts grown on conventional culture plates at CTB and STB stages. Surprisingly, apoA-1-mediated cholesterol efflux operated almost exclusively at the apical-maternal side, where ABCA1 was also localized by immunofluorescence. We found greater cholesterol efflux capacity in STB, which was increased by liver-X-receptor agonist treatment and decreased by ABCA1 inhibition. We conclude that at term the apoA-1/ABCA1 pathway is rather involved in cholesterol transport to the mother than in transfer to the fully developed fetus.

## 1. Introduction

Cholesterol is a crucial component of the biological membranes of all mammals, being involved in maintenance of cell integrity and cellular functions [1]. It is also a precursor for the synthesis of steroid hormones (e.g., progesterone and prednisolone), which are, amongst others, critically important for pregnancy maintenance and/or maturation of fetal organs, such as the lung [2,3,4]. During pregnancy, the mother adapts her secretory activities to maintain a delicate balance between her own physiological needs and the requirements of the fetus for embryogenesis, organogenesis, or late maturation. An increase in circulating maternal cholesterol occurs in the third trimester, when fetal maturation takes place [5]. In addition, investigations in mice showed that lung hypoplasia and delayed vascular development in late gestation are associated with insufficient maternal cholesterol delivery [3]. Thus, understanding the mechanisms of cholesterol delivery to the fetus is not only important during the first trimester in terms of embryogenesis, but also throughout the third trimester in the context of complemental fetal growth and maturation of all organ systems indispensable for immediate survival after birth.

Overall, the delivery of maternal cholesterol to the fetus depends on the placental abundance of cholesterol transporters and receptors. ATP-binding cassette (ABC) transporter proteins are cellular transmembrane proteins that mediate lipid efflux from cells to lipoprotein particles. Two major members of the ABC transporter family, ABCA1 and ABCG1, were shown to be implicated in cellular cholesterol transfer to lipid poor apolipoprotein (apo)-A1 and high-density lipoprotein (HDL), respectively [6,7,8]. During the transport process across the placenta, cholesterol has to pass physiological barriers separating the maternal from the fetal circulation. These barriers consist of the syncytiotrophobast layer (STB), a multinucleated layer of fused precursor cytotrophoblasts (CTB) facing the maternal circulation, and the fetal endothelium, composed of endothelial cells, localized at the basal (fetal) side of the STB [9,10].

Several studies have identified ABCA1 and ABCG1 in placental tissues and cells at various cellular localizations [11,12,13,14]. Further investigations revealed that only the abundance of placental ABCA1 (but not ABCG1) exhibited dependence on the gestational age [14], supporting the involvement of ABCA1 with adequate in utero development of the fetus. In support of this assumption, a decrease of placental *ABCA1* expression and cholesterol transport activity was observed in the gestational disease pre-eclampsia [14,15]. Moreover, deletion of *ABCA1* in mice led to an approximately 30% decrease in transfer of maternal cholesterol to the fetus, while suppression of *ABCG1* did not affect the transfer process [16]. A recent study in humans revealed also a highly significant correlation between maternal *ABCA1* genotypes and the severity in Smith-Lemli-Opitz syndrome (SLOS) [17]. The treatment of pregnant female mice with an agonist of the liver-X-receptor (LXR) transcription factor has been suggested as a potential therapeutic treatment of SLOS [16,18]. LXR is a key regulator of the expression of multiple genes implicated in cholesterol homeostasis and transport such as *ABCA1*, sterol-response element binding protein 2 (*SREBP-2*), scavenger receptor B1 (*SR-B1*) [19,20,21,22,23], or low-density lipoprotein (LDL) receptor (*LDL-R*). SR-B1 is involved in both inward and outward transport [24], while LDL-R mediates inward cholesterol uptake from lipoprotein particles [25].

To date, the directionality and underlying regulation of apoA-1 mediated transplacental cholesterol transport is not fully elucidated. Thus, the objectives of this study were to compare (1) the expression of selected cholesterol homeostatic genes, and (2) the orientation of the apoA-1 mediated cholesterol efflux in a highly physiological model [26], namely primary trophoblast cells isolated from human term placentas. The isolated trophoblasts were tested before and after in vitro differentiation, the latter being characterized by spontaneous formation of a functional syncytium in vitro. We hypothesized that (1) cholesterol transport capabilities are increased in differentiated (STB) as compared to undifferentiated (CTB) trophoblasts, and (2) cholesterol efflux to apoA-1 is predominantly oriented towards the basal (fetal) side of the STB to support the high fetal demands and fetal organ maturation close to birth.

## 2. Results and Discussion

The present study discusses maternal–fetal cholesterol transport in and across the placenta at term when the maturation of fetal tissues and organs occurs. The study elicits the cholesterol transport capacities of trophoblasts in relation to their differentiation state and provides new insights into the directionality of the apoA-1/ABCA1-mediated cholesterol transport at term.

### 2.1. Characterization of Isolated Trophoblasts and Efficiency of In Vitro Differentiation

The purity of isolated primary CTB from tissue specimens is critically important, and potential cellular contamination might influence the interpretation of in vitro studies [27]. In the current study, trophoblasts were isolated following the previously published isolation protocol [13], complemented by a detailed routine evaluation of cellular identity and physiological features.

#### 2.1.1. Cell Morphology

**Figure 1:**
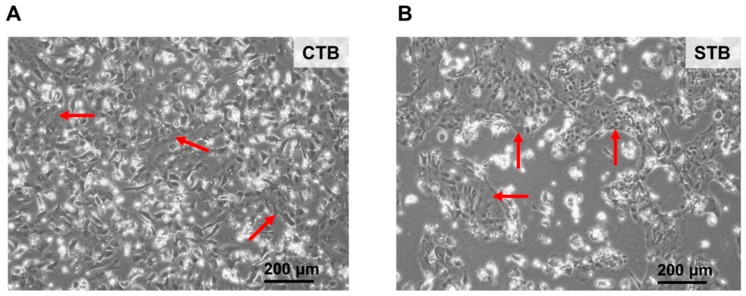
Morphology of trophoblast cells. (**A**) Primary undifferentiated cytotrophoblasts (CTB) were cultured as described in Materials and Methods. After 24 h cultivation cells show characteristics of mononucleated epithelial cells with well-demarcated cell borders and large nuclei as highlighted by the red arrows; and (**B**) after 3 days of cultivation CTB fused to form multinucleated entities representing a syncytium (STB) as indicated by the red arrows. STB appear as a single continuous layer without cell borders. Bright field images were captured at 10× magnification. Trophoblasts were isolated from a healthy human placenta at term.

Freshly isolated primary cells appeared like single nucleated and cuboid cells with well-demarcated cell borders, corresponding to trophoblasts (CTB) [13] (Figure 1, left panel). After 3-days of cultivation CTB have fused and formed multinucleated cellular entities representing the syncytium (Figure 1, right panel). These cells appear as a continuous layer without intercellular boundaries, and form island-like shapes when grown on conventional culturing plates. In general, syncytium (STB) formation starts already 24 h after cultivation [13], and STB maintain their morphological characteristics over several days.

#### 2.1.2. Protein Expression of Specific Cell Type Markers

**Figure 2:**
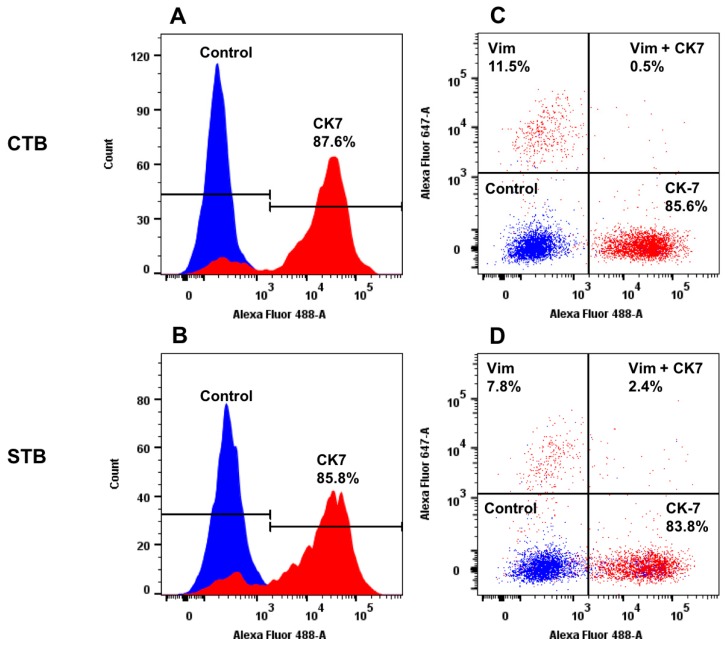
Protein expression of cell-specific markers in CTB and STB cultures. Protein expression of cytokeratin (CK) 7 and vimentin (vim), specific markers for epithelial cells and mesenchymal cells, respectively. (**A**–**D**): Representative protein expression patterns of CK7 and vim in isolated trophoblasts after 24 h (undifferentiated trophoblasts; CTB, panel (**A**) and (**C**)) and 72 h (differentiated trophoblasts; STB, panel (**B**) and (**D**)) of cultivation was assessed by flow cytometry. Specific staining of CK7 and vimentin is depicted in red; the isotype control staining is shown in blue. The images are shown as overlays on isotype control. The black lines on panels A and B indicate the positive (CK-7) and negative (isotype control) expression patterns. Data were evaluated by using FlowJo data analysis software.

The purity of CTB and STB was evaluated by analyzing the cellular expression of cytokeratin (CK)7, vimentin, von Willebrand factor (vWF), and E-cadherin by flow cytometry. CK7 and E-cadherin have been chosen, because they are commonly used markers for epithelial cells [28,29]. We also assessed to which extent potential cellular contamination by mesenchymal cells or endothelial cells occurred and/or have been propagated with cultivation. To this end, the expression of vimentin and vWF was analyzed to evaluate the presence of mesenchymal and endothelial cells, respectively [28,29,30]. As shown in Figure 2B (left and right panels), there were clear increases in fluorescence as compared to the isotype controls, indicating that both CTB and STB express high levels of CK7. The degree of CK7 positivity in CTB and STB was above 85% (Figure 2A,B), suggesting high purity and stability of the trophoblast cultures. Additional purification by negative immuno-magnetic selection [13] was not performed due to the lack of substantial improvement of purity.

The cellular costaining for CK7 and vimentin positivity confirmed the predominantly epithelial character of the isolated cells: CK-positivity ranged from ~84 to 86%, while vimentin-positivity was between ~8 and 12% (Figure 2C,D). These data indicated the absence of significant mesenchymal contaminations and excluded the propagation/overgrowth of contaminating cells during cultivation. Similarly, the results of the costaining for E-cadherin and vWF positivity in CTB and STB confirmed the epithelial character of the isolated and cultivated cells. The predominantly negative staining for vWF, ranging from 0.37 to 1%, illustrates the negligible contamination by endothelial cells, supporting the homogeneity of the cell isolation. In summary, CTB and STB were pure and stable throughout the experimentally applied culture conditions and time, allowing an unbiased interpretation of the consecutive in vitro cholesterol transport assays.

#### 2.1.3. Secretion of Syncytial Factors

**Figure 3:**
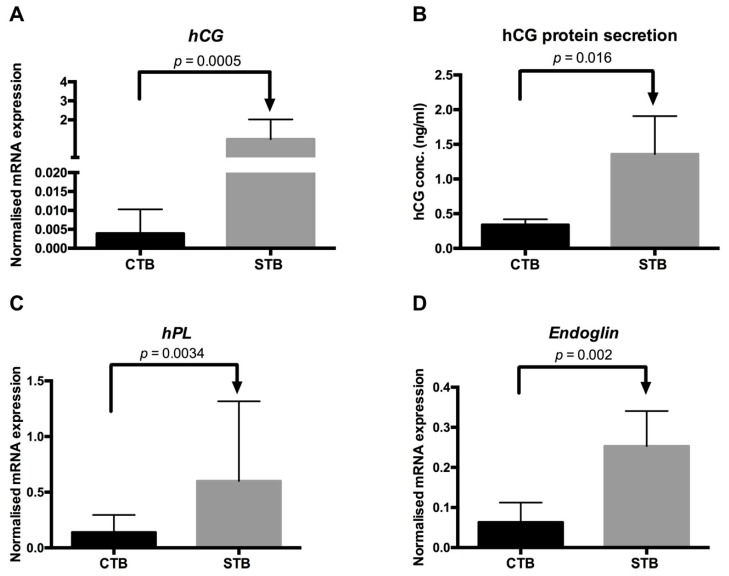
Expression of syncytial markers. (**A**,**B**) The mRNA abundance and protein secretion of human chorionic gonadotropin (*hCG*) in primary undifferentiated (CTB) and differentiated (STB) trophoblast cells. The gene expression of *hCG* was assessed by real-time RT-PCR and normalized to the reference gene as described in Materials and Methods [36]. Primary trophoblasts were isolated from healthy term placentas as described in Materials and Methods. Data are shown as mean ± SD of 12 independent experiments measured at least in duplicates. The protein secretion of hCG in CTB and STB was measured by using a human hCG ELISA kit following the manufacturer’s instructions. Data are shown as mean ± SD of at least three independent experiments; and (**C**,**D**) The mRNA abundance of *human placental lactogen* (*hPL*) and *endoglin* in CTB and STB was evaluated as described above for *hCG*. Data are shown as mean ± SD of three independent experiments measured at least in duplicates.

In a second step, we analyzed whether CTB and STB exhibit physiological features similar to in vivo conditions [31,32,33]. Therefore, we compared the gene expression and secretion of human chorionic gonadotropin (*hCG*), human placental lactogen (*hPL*), and *endoglin* between CTB and STB. The abundances of *hCG* and *hPL* were determined because it has been demonstrated that the expression of these genes increases with trophoblast differentiation and syncytium formation [31,33]. Endoglin is strongly expressed on the apical membrane of STB with a possible role in syncytial invasiveness and angiogenesis [32,34]. Concerning *hCG*, we found a markedly higher mRNA gene expression in STB (*p* < 0.001 and *p* < 0.05, respectively) as compared to CTB (Figure 3A).

Consistently, hCG protein secretion was greater in STB than CTB (Figure 3B), mirroring the mRNA gene expression data. These data indicate that STB exhibit in vivo-like characteristics, suggesting that our model is suitable to mimic in vivo processes occurring in the placenta. There was a high correlation between *hCG* mRNA and protein release (*r* = 0.7; *p* = 0.0082), in agreement with data reported by others [35]. This suggests that *hCG* mRNA expression per se is a good indicator of trophoblast differentiation in an experimental setup where protein data are unavailable.

Moreover, we found that *hPL* (Figure 3C) and *endoglin* (Figure 3D) mRNA levels were higher in STB (*p* < 0.005 and *p* < 0.005, respectively) than in CTB. Considering that endoglin is involved with trophoblast differentiation in the invasive pathway [32,34], the increased mRNA expression in STB as compared to CTB confirms the successful in vitro trophoblast differentiation and syncytium formation. In conclusion, the investigated trophoblast cells represented a highly physiological model and were suitable for the study of cholesterol transport, including directionality, in and/or across the placenta.

### 2.2. Cholesterol Transport in CTB and STB

Considering that discrepancy in cholesterol transport capacities may exist between CTB and STB, we compared their expression profiles for *SR-B1, LDL-R, ABCA1, SREBP-2*, and *apoA-1*, as well as their cholesterol transport activity (inward and outward) to identify potential differences and compensatory mechanisms. These comparisons were also performed in cells treated with T0901317, an agonist of LXR, because this nuclear receptor is a key regulator of many genes implicated in cellular cholesterol homeostasis [21,37].

#### 2.2.1. Gene Expression Studies

We determined the mRNA expression of *ABCA1*, responsible for cellular cholesterol efflux [12,13], and of *SR-B1* and *LDL-R*, which are associated with the bidirectional transport and uptake of cholesterol, respectively [24,38]. As the placenta is a steroidogenic tissue, we also assessed in CTB and STB candidate genes representing either cholesterol synthetic capacities (*SREBP-2*) or autocrine and paracrine pathways regulating cholesterol transport (e.g., by apoA-1-mediated effects). It is well known that diverse effector proteins (e.g., hormones) released by a secretory cell can either act directly on itself and regulate cellular functions (autocrine effect) or can affect the neighboring cells (paracrine effect). We hypothesized that similar autocrine and paracrine effects by endogenous apoA-1 may occur in the context of cholesterol transport regulation in trophoblast cells. The primers used for the amplification of target genes are listed in Table 1.

Our data showed no statistically significant difference in *SR-B1* mRNA expression between CTB and STB at steady state conditions (i.e., without additional stimulation; *p* = 0.50), while an increased expression (*p* = 0.041) was observed in cells treated with the LXR agonist T0901317 (Table 2).

The differential abundance of *SR-B1* mRNA between CTB and STB was observed only after LXR induction by T0901317, because the *SR-B1* gene contains an LXR response element [41] supposedly potentiating differences between the two cell stages. These results suggested also marked differences in SR-B1 mediated cholesterol uptake between CTB and STB after exposure to an LXR agonist, which indeed occurred (Table 2, see also below). Interestingly, the gene expression of *LDL-R*, another cholesterol uptake gene, was markedly increased in CTB (*p* = 0.068) and STB (*p* = 0.0078) after treatment with T0901317 (Table 2). There was no differential abundance of *LDL-R* between CTB and STB neither before nor after treatment. This suggests a universal role of LDL-R in cholesterol uptake of trophoblast cells independent of their differentiation stage.

On the other hand, we found that the *ABCA1* mRNA level was significantly higher in STB than CTB (Table 2), suggesting a greater cholesterol efflux capacity in STB. Consistent with data published elsewhere [20,42], *ABCA1* mRNA was significantly induced (by a factor of approximately 10 in the current study) after LXR agonist treatment both in CTB and STB (Table 2). In summary, the increased *SR-B1* and *ABCA1* mRNA levels in STB as compared to CTB may predict that increased cholesterol uptake by cells is accompanied by enhanced cholesterol release through an ABCA1-dependent mechanism.

To get insights into the associations between cholesterol uptake, endogenous cholesterol synthesis, and cholesterol release, we also compared the mRNA expression levels of *SREBP-2* between CTB and STB. SREBP-2 is a transcription factor that upregulates the endogenous cholesterol synthesis and increases cholesterol uptake in response to cholesterol deficiency [22]. Our data did not show differential *SREBP-2* mRNA expression patterns between CTB and STB independent of LXR stimulation. This predicts that SREBP-2-dependent compensatory mechanisms (for instance in CTB) are not prominently involved in the differential *SR-B1* mRNA expression between CTB and STB (Table 2). Furthermore, our data showed a significant response to LXR agonist treatment, resulting in an increase of *SREBP-2* expression in both CTB and STB. This is in contrast to previous findings reporting a lack of LXR promoting effects on *SREBP-2* mRNA expression in trophoblast cells [42]. This discrepancy might be due to the high variability reflected by the high standard deviations of *SREBP-2* mRNA expressions documented in that study.

Concerning the potential autocrine and/or paracrine regulation of cholesterol transport, we found that *apoA-1* mRNA expression levels did not vary between CTB and STB, and were also unaffected by treatment with the LXR agonist (Table 2). In this context, our own observations also revealed that STB secrete tendentially more apoA-1 (1.6-fold; *n* = 3) than CTB confirming the corresponding *apoA-1* mRNA data.

#### 2.2.2. Cholesterol Uptake: Inward Transport

In the next step, we compared cholesterol uptake rates in CTB and STB (Table 2). We assumed that inward cholesterol transport capacities are critically important for the transport of maternal cholesterol to the fetus when fetal maturation occurs. In the current study, we observed, at steady-state conditions, a trend towards greater cholesterol uptake rates in STB as compared to CTB (Table 2). The uptake rates were significantly enhanced after exposure to the LXR agonist and showed a significant difference between STB and CTB (Table 2). These data indicate differential inward cholesterol transport rates of trophoblasts, dependent on their differentiation state. Considering the similar *SREBP-2* gene expression between CTB and STB, the difference in the uptake rates does not seem to be related to differential intrinsic SREBP-2 dependent cellular mechanisms. In this regard, we found significant correlations between *SREBP-2* mRNA abundance and cholesterol uptake in both CTB and STB at steady state conditions (Table 2). These findings confirm the importance of the SREBP-2 pathway in regulating cholesterol transport processes in trophoblasts.

We further observed markedly increased inward cholesterol transport rates in both CTB and STB after treatment with the LXR agonist (Table 2). This finding is consistent with other studies [16,43], which reported that LXR enhances maternal cholesterol delivery to the fetus. Thus, our data confirm the role of SR-B1 as an LXR-dependent mechanism controlling inward cholesterol uptake rates. This mechanism is still important at term pregnancies, because the maternal cholesterol delivery is required for covering fetal demands. Impaired fetal maturation and neonatal health were observed in case of disturbed maternal cholesterol delivery during late gestation [3]. In the current study, we did not find statistically significant correlations between cholesterol uptake rates *and SR-B1* expression in STB exposed to an LXR agonist (Table 2). However, this correlation was seen in unstimulated STB (Table 2). This apparent discrepancy could be due to the fact that cholesterol uptake rates are not exclusively driven by SR-B1 [24,38]. In this context, we also observed that cholesterol uptake was significantly correlated with *LDL-R* in CTB treated with LXR-agonist (Table 2).

#### 2.2.3. Cholesterol Efflux: Outward Transport

Next, we evaluated the outward directed cholesterol transport and focused herein on the apoA-1 mediated efflux, i.e., ABCA1-dependent transport mechanism for the following reasons: Firstly, a very recent study carried out in primary trophoblasts indicated that increased ABCA1 protein levels led to an elevation of cholesterol efflux, while a reduction in ABCA1 resulted in suppressed cholesterol efflux [20]. Secondly, previous studies from us, and others, have reported that, despite of the localization of both ABCA1 and ABCG1 in placental tissues and cells [11,12,13], only placental *ABCA1* expression (but not ABCG1) exhibits a gestational-age-dependent expression pattern [14], suggesting a key role of this protein in transplacental cholesterol transport during gestation.

**Figure 4:**
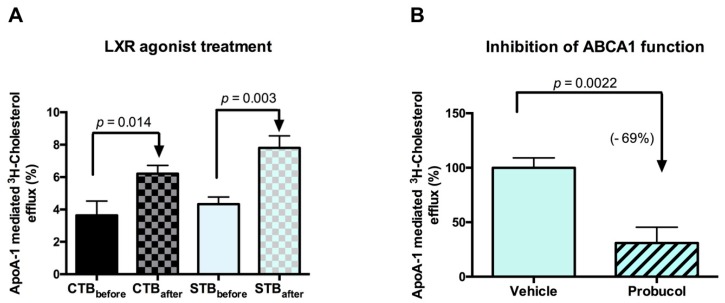
Stimulation and inhibition of ApoA-1 mediated cholesterol efflux. (**A**): ApoA-1 mediated cholesterol efflux was measured before and after cell treatment with T0901317, a synthetic agonist of the liver X receptor (LXR). 0.1 µM T0901317 was applied to the cells throughout the entire experiment as described in Materials and Methods. Data are shown as mean ± SD of at least three independent experiments. (**B**): Inhibition of apoA-1 mediated cholesterol efflux in STB using an ABCA1 inhibitor. Probucol (10 µM) was applied to cells during the entire experiment. Procedures for cholesterol efflux and calculations were as described in detail in Materials and Methods. All data are mean ± SD of three independent experiments.

In the current study, we found a markedly higher level of apoA-1 mediated cholesterol efflux in STB than in CTB (*p* = 0.0068) after LXR agonist treatment. On the other hand, in both CTB and STB, the apoA-1 mediated efflux was significantly promoted by the LXR agonist (Figure 4A), consistent with data reported by others [20]. The increase of ABCA1-mediated cholesterol efflux (~up to 2-fold) was less pronounced in comparison with the magnitude of *ABCA1* mRNA upregulation (~up to 10-fold). A similar relationship between *ABCA1* mRNA expression and apoA-1 dependent efflux was previously reported by others in trophoblasts [44] and in human fetoplacental endothelial cells [43].

In addition, as shown in Figure 4B, the major implication of ABCA1 in apoA-1 mediated efflux in trophoblast cells was demonstrated. Indeed, there was a drastic suppression of cholesterol efflux observed in STB in the presence of probucol, an inhibitor of ABCA1 function [45]. A similar effect of probucol was observed in CTB. These findings confirm the prominent role of the apoA-1/ABCA1 pathway in mediating cholesterol transport.

### 2.3. Directionality of the Cholesterol Transport and ABCA1 Localization

**Figure 5:**
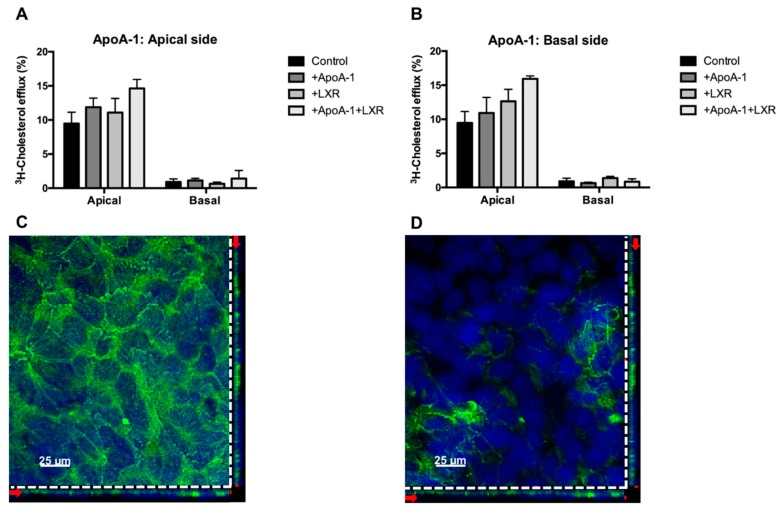
Transport activity and localization of ABCA1 in human primary trophoblast cells polarized on Transwell^®^ system. (**A**,**B**) The confluent trophoblast monolayer was loaded with 1 µCi of ^3^H-cholesterol at the apical side for 24 h. After equilibration, 10 µg/ml of apoA-1 was added to the apical panel (**A**) or the basal side panel (**B**) for 8 h. Data are shown as mean ± SD from a representative experiment. Details of the efflux procedure are described in Materials and Methods, and (**C**,**D**) representative immunofluorescence staining of ABCA1 in the confluent STB monolayer. The orthogonal projections through the Z-stack suggested that ABCA1 (**green**) is predominantly expressed at the apical side (Figure 5C) while it was barely detectable at the basal side (Figure 5D) of the STB monolayer. Nuclei were counterstained with 4’,6-diamidino-2-phenylindole (DAPI, **blue**). The horizontal and vertical red arrows on the orthogonal plane show the level of the selected stack. The upper level of the stack shown by the red label on the right corner of the image (Figure 5C) corresponds to the apical outer membrane. The bottom level stack marked by the red label on the right corner of the image (Figure 5D) corresponds to the basal outer membrane of the STB. Details of the staining procedure are described in Materials and Methods.

The importance of the apoA-1/ABCA1 pathway in mediating the transplacental cholesterol transport is well-documented [11,12,14]. However, it still remains unclear whether, or to what extent, this pathway is involved in dispatching maternal cholesterol to the developing fetus close to parturition time. Therefore, the directionality of the apoA-1/ABCA1-mediated outward cholesterol transport was analyzed, using the Transwell^®^ system with an STB monolayer derived from term human primary trophoblast cells. This cell model has already been thoroughly validated as a useful system to study transplacental nutrient transport [46]. Unexpectedly, we found that apoA-1/ABCA1-mediated cholesterol efflux occurred predominantly at the apical (maternal) side, whereas only minor transport could be detected at the basal side of STB (Figure 5A).

The herein reported predominantly apically directed cholesterol transport argues against the hypothesized role of apoA-1/ABCA1 in the delivery of maternal cholesterol to the developing fetus. In this context, it is important to note that the biophysical and biochemical properties of the STB monolayers in the current study were similar to those previously reported [46]. Consistent with the observed cholesterol transport orientation, the *Z*-stack confocal image analysis showed markedly higher ABCA1 protein expression at the apical outer membrane (Figure 5C) than at the basal side (Figure 5D) of the STB.

Taken together, these data imply that, at term, the apoA-1/ABCA1 pathway predominantly acts in transporting cholesterol back to the maternal circulation. To the best of our knowledge, this has not been demonstrated so far in a physiological system such as primary term human trophoblasts. Indeed, the successful establishment of a confluent monolayer with primary trophoblasts isolated from term placenta has only recently been achieved [46]. Until then, cell-based vectorial cholesterol transport studies using primary trophoblast cells have been considered as unrealistic [44] and maternal–fetal cholesterol transport processes were mainly investigated in BeWo cells, a first trimester derived choriocarcinoma cell line. These cells have been described to express, in comparison with differentiating trophoblasts, significantly lower abundances of ABC transporters such as the multidrug resistance protein (MDR) 1/P-glycoprotein (P-gp), the P-gp homolog MDR3, breast cancer resistance protein, and multidrug resistance proteins 1 and 2 [47]. However, the comparative expression patterns of ABCG1 and ABCA1 were not reported in this study [47]. Other investigations demonstrated a greater protein expression level of ABCG1 in BeWo as compared to placenta tissues [48], but again ABCA1 expression was not shown. A study investigating vectorial cholesterol transport in BeWo cells failed to demonstrate the efflux of cholesterol to apoA-1 at the basal (fetal) side [49]. However, basally oriented transport was observed when fetal human serum or phospholipid vesicles were used as acceptors. Despite the apparent importance of ABCA1-mediated cholesterol efflux, no ABCA1 mRNA or protein expression data were reported [49]. Of note, BeWo cells are derived from a first trimester placenta. Therefore, caution might be required in the direct comparison of the apoA-1-mediated cholesterol transport data obtained in BeWo cells (indicating lack of cholesterol efflux to apoA-1 at the fetal side) and the present findings in term placentas (demonstrating predominantly apically, i.e., maternally oriented cholesterol transport).

The underlying physiological reasons for the transport orientation seen in the present study have not been investigated so far. However, it is tempting to speculate that the placenta acts in this way to protect the fetus from excess cholesterol transfer. Indeed, there are reports suggesting that excess cholesterol can promote the formation of atherosclerotic plaques in the fetus, thereby programming the occurrence of metabolic diseases later in life [50,51]. Alternatively, the transport orientation towards the mother could also serve to avoid cholesterol accumulation in the placenta, thereby reducing the risk of increased endogenous steroid hormone secretion and concomitant harmful fetal exposure. In this regard, it is known that antenatal exposure to excess steroid hormones is harmful for the fetus. It leads to decreased birth weight and increased vulnerability of the fetus to develop serious diseases later in life [52,53]. Since data reported here are obtained exclusively from term placentas, it cannot be excluded that the directionality of the apoA-1/ABCA1 mediated cholesterol transport is reversed at early stages of pregnancy, when the fetus is fully dependent on maternal cholesterol supply. To clarify this aspect, transport studies with trophoblast cells isolated from first trimester placentas are required.

## 3. Materials and Methods

### 3.1. Human Placental Tissue Collection

Placental tissues were collected after obtaining informed consent from pregnant women at the Division of Gynecology and Obstetrics of the Lindenhofgruppe, Bern, Switzerland. The study was conducted in accordance with the Declaration of Helsinki, and the protocol was approved by the Ethics Committee of the Canton of Bern (Basec Nr. 2016-00250; 14/04/2016). Term placentas (38–40 weeks; *n* = 17) were collected from uncomplicated pregnancies after elective caesarean section without prior labor symptoms upon patients’ request or due to breech presentation.

### 3.2. Isolation of Primary Trophoblasts and Cell Culture

Villous trophoblast cells were isolated from term placental tissue by enzymatic digestion and Percoll gradient separation, as previously described [13] with minor modifications. In brief, approximately 50 g of villous tissue was washed in 0.9% NaCl (Sigma, Saint Louis, MO, USA) four times for 5 min. Thereafter the tissue was minced and digested four times with 0.25% trypsin (Sigma, USA) and 300 IU/mL deoxyribonuclease I (Sigma, Saint Louis, MO, USA) at 37 °C (20 min each). The cell suspension was filtered and overlayed on fetal bovine serum (FBS, Seraglob, Schaffhausen, Switzerland). After centrifugation at 1000× *g* for 15 min at 10 °C, the cell pellet was collected in Dulbecco’s modified Eagle’s medium containing 4.5 g/L glucose (DMEM-HG, Gibco, Paisley, UK) basic medium (without FBS) and filtered through 100 µm strainer (BD Biosciences, Durham, NC, USA). Next, cells were overlayed on a discontinuous Percoll^®^ (Sigma, Saint Louis, MO, USA) density gradient. After centrifugation, CTBs were located at the layer corresponding to 1.046–1.065 g/mL (35% to 50%) density [26]. The isolated cells were cultured at a density of 0.2 × 10^6^ cells/cm^2^ and 0.5 × 10^6^ cells/cm^2^ in 6-well or 24-well CellBIND plates (Costar, Kennebunk, ME, USA), respectively, using complete DMEM-HG medium (including 10% FBS and 1× antibiotic-antimitotic (Gibco, Grand Island, NY, USA).

### 3.3. Cell Morphology

Cultured CTB on 24-well CellBIND plates were inspected by microscopy. After 24, 48, and 72 h of cultivation bright-field images of CTB and STB were captured using a Leica DMi1 inverted microscope (Leica, Wetzlar, Germany). The images were processed with the corresponding Leica Software to compare changes in cell morphology between day 1 and 3.

### 3.4. Flow Cytometry Analysis of Cell Purity

The purity of CTB and STB was evaluated by staining the trophoblast cells with specific cell markers followed by flow cytometry analysis. Cells were grown on CellBIND plates, detached by accutase (Sigma, Saint Louis, MO, USA), and fixed with 4% formaldehyde (Thermo Scientific, Rockford, IL, USA) for 10 min on ice. After washing with Dulbecco’s phosphate buffered saline (DPBS; Sigma, Saint Louis, MO, USA), cells were centrifuged at 200× *g* for 10 min at 4 °C and then permeabilized with 0.5% Tween-20 (*w*/*v*) (Sigma, Saint Louis, MO, USA) in DPBS for 15 min at room temperature. For evaluation of cell purity, dual staining of CTB and STB with directly labeled antibody cocktails, prepared in staining buffer (5% FBS, 0.1% Tween-20 (*w*/*v*) in DPBS), has been performed. Antibody cocktails contained either anti-CK7, labeled with Alexa Fluor 488^®^ (Novus Biologicals, Littleton, CO, USA) plus anti-vimentin labeled with Alexa Fluor 647^®^ (Novus Biologicals, Littleton, CO, USA) or anti-E-cadherin, labeled with Alexa Fluor 488^®^ (Novus Biologicals, Littleton, CO, USA) plus anti-vWF labeled with Alexa Fluor 647^®^ (Novus Biologicals, Littleton, CO, USA). Cells were incubated with the respective antibody cocktail for 45 min on ice, followed by 2 times washing (1 min each). After centrifugation at 200× *g* for 10 min at 4 °C, pelleted cells were suspended in DPBS and acquired by flow cytometry (BD FACS LSRII; BD Biosciences, San Jose, CA, USA). Data acquisition and analysis for each staining was based on at least 10,000 events and performed by using BD FACSDiva™ (BD Biosciences, San Jose, CA, USA) and FlowJo^®^ software version-10 (FlowJo LLC, Ashland, OR, USA). Since CTB and STB are epithelial cells, positive staining for CK7 and E-cadherin served as positive cell markers [28,29]. On other hand, vimentin, known to predominantly stain mesenchymal cells, fibroblast, and stromal cells [28,29,30], and vWF selectively staining endothelial cells, served as negative markers for CTB and STB. These staining procedures allowed the assessment and quantification of potential cellular contaminations by mesenchymal cells, fibroblasts, stromal cells, and endothelial cells, respectively.

### 3.5. Quantitative RT-PCR

Quantitative RT-PCR was performed as described previously [54]. Total RNA was extracted using Trizol reagent (Invitrogen, Grand Island, NY, USA). RNA concentration was quantified with the NanoDrop 1000 (Thermo Fisher Scientific, Wilmington, DE, USA) by measuring the absorbance at 260 nm. Additionally, the OD 260/230 and the OD 260/280 ratio showing RNA purity were determined.

Varying amounts of total RNA were then reverse transcribed to cDNA with the GoScript™ Reverse Transcriptase System (Promega, Madison, WI, USA) according to the manufacturer’s instructions. RT-PCR was performed on a ViiA^™^7 RT-PCR System by using SYBR^®^ Green PCR master mix detection kit (Promega, Madison, WI, USA). The mRNA expression of selected syncytial markers, namely *hCG*, *hPL*, and *endoglin*, was compared between day 1 and 3 and served as markers confirming STB formation. *SR-B1*, *ABCA1*, *apoA-1*, and *SREBP-2* represent important components of cellular cholesterol uptake, transport, and cholesterol homeostasis. *Tyrosine 3-monooxygenase/tryptophan 5-monooxygenase activation protein, zeta polypeptide* (*YWHAZ*) was selected as endogenous reference gene [14]. All quantitative PCR experiments were performed with nontemplate negative controls and measured on the ViiA™ 7 Real-Time PCR System (Applied Biosystems, Carlsbad, CA, USA). The gene expression of targets was calculated by using 2^−^^Δ*C*t^ method, as previously described [36]. Characteristics of the target genes and the applied primers are listed in Table 1.

### 3.6. ELISA for hCG Secretion

The secretion of hCG protein into CTB and STB culture media was measured by using human hCG (intact) ELISA kit (Sigma, Saint Louis, MO, USA) following the manufacturer’s instructions. In brief, hCG standards, negative controls, and culture medium samples, were loaded onto hCG pre-coated wells and incubated for 2.5 h at room temperature. After four times washing, biotinylated detection hCG antibody was added and incubated at room temperature for 1h with gentle shaking. Thereafter, the wells were washed three times, followed by incubation with HRP-streptavidin solution for 45 min at room temperature with gentle shaking. The wells were subjected to three additional washing cycles before adding the ELISA colorimetric TMB (3,3’,5,5’-tetramethylbenzidine) reagent. After 30 min incubation at room temperature in the dark, the reaction was stopped by adding stop solution provided in the kit. Consecutive absorbance measurements were carried out at 450 nm on a *V*max microplate reader (Molecular Devices, San Jose, CA, USA). The concentrations of hCG released by CTB and STB were extrapolated from the slope of the standard curve.

### 3.7. Cholesterol Transport Studies

The main objective of this study was to evaluate cholesterol uptake (i.e., inward transport) and efflux (outward transport) rates in CTB and STB. This was investigated by applying two different experimental models, namely trophoblast cells grown on conventional culture plates and confluent human primary trophoblast monolayers using pre-coated polycarbonate inserts (Transwell^®^ system).

#### 3.7.1. Cholesterol Transport in Conventional Plates

Freshly isolated CTB were seeded at a density of 0.5 × 10^6^ cells/cm^2^ in CellBIND 24-well plates and allowed to attach for 8 h. Then cells were loaded for 24 h with 1μCi/mL ^3^H-cholesterol (PerkinElmer, Boston, MA, USA). For cholesterol transport studies in STB, cells were first allowed to form multinucleated cell patches (approx. 72h) before being loaded with 1 µCi/mL ^3^H-cholesterol. After 24 h loading, cells were equilibrated for 4 h in serum-free DMEM HG medium. Cholesterol uptake rates were calculated by relating the amounts of radioactivity remaining in the medium to the initially loaded radioactivity. The cholesterol efflux period was initiated by adding 10 μg/mL of apoA-1 (Sigma, Saint Louis, MO, USA), in serum-free DMEM HG medium for 2, 4, 6, and 8h. After collecting the efflux medium, the plates were frozen at −20 °C for 30 min. The details used for lysate collection and cholesterol efflux measurements were as previously described [55]. ApoA-1 mediated cholesterol efflux was obtained by subtracting the efflux value in the absence of apoA-1 from that in the presence of apoA-1 [55]. Where applicable, cells were exposed to 0.1 μM T0901317 (synthetic LXR agonist; Sigma, Saint Louis, MO, USA) or 10 μM probucol (ABCA1 inhibitor; Sigma, Saint Louis, MO, USA) throughout the entire experiment.

#### 3.7.2. Cholesterol Transport in Transwell^®^ System

Our final goal was to determine the directionality of the apoA-1 mediated cholesterol efflux using STB monolayers grown on Transwell^®^ inserts (Falcon, Durham, NC, USA) consisting of apical (maternal-like) and basal (fetal-like) compartments. For determining ^3^H-cholesterol uptake and efflux, 12 well Transwell^®^ inserts with 0.4 μm pore PET membrane were used which were coated with Matrigel (BD Biosciences, Bedford, MA, USA) at a concentration of 25 µg/cm^2^ prior to the experiment. The procedures used for ^3^H-cholesterol loading, uptake, and efflux determination were similar to those described for conventional plates with the exception that 1 × 10^6^ cells/cm^2^ were seeded. The cholesterol transport studies were performed on day 7, after having validated both the tightness and barrier integrity of the STB monolayer, as previously described [46]. In brief, trans-epithelial electrical resistance (TEER) was measured daily using a Millicell ERS-2 Volt-Ohm Meter (Millipore, Bedford, MA, USA), as previously described [46]. The TEER value was determined on the Matrigel-coated inserts in the presence or absence of trophoblast cells. In addition, the monolayer permeability properties were evaluated by measuring daily the passage of the fluorescence dye Lucifer yellow (LY; Sigma, Saint Louis, MO, USA) across the trophoblast monolayer as previously described in detail [46].

### 3.8. Immunofluorescent Confocal Microscopy

In addition to determining the biophysical and biochemical properties of the trophoblast monolayer, the localization of ABCA1 was analysed using confocal microscopy, as previously described [46]. In brief, the confluent trophoblast monolayers grown on Transwell^®^ membranes were first fixed in methanol at −20 °C for 30 min, and then washed in 0.1 M glycine (Merck, Darmstadt, Germany) for 15 min. After blocking with a cocktail of 2% bovine serum albumin (Sigma, Saint Louis, MO, USA) and 4% goat serum (Sigma, Saint Louis, MO, USA) for 1 h at room temperature, they were incubated with anti-ABCA1 antibody (Novus Biologicals, Littleton, CO, USA) overnight at 4 °C. After washing, the mixture of goat anti-rabbit (H + L) Alexa Fluor 488 (Invitrogen, Eugene, OR, USA) with 4’,6-diamidino-2-phenylindole (DAPI; Sigma, Saint Louis, MO, USA) was added for 1 h at room temperature in the dark. Membranes were mounted on glass slides using Mowiol mounting media (Sigma, Saint Louis, MO, USA). The immunofluorescent staining was examined using a Nikon C1 confocal system (Nikon, Tokyo, Japan) equipped with a 40× S Fluor oil-immersion objective. Images were acquired and optimized with EZ-C1 software (Nikon, Tokyo, Japan).

### 3.9. Statistics

Data analysis and statistical evaluation were performed with GraphPad Prism^®^ software (GraphPad software, La Jolla, CA, USA). All data are expressed as mean ± SD. Differences in the mRNA expression, and in cholesterol transport activities between CTB and STB as well as the effects of treatment were analyzed by using unpaired and paired *t*-tests, respectively. The correlation coefficients between cholesterol transport and gene expression as well as between mRNA and protein levels of hCG were evaluated by using the Pearson correlation test. The level of statistical significance was set at *p* < 0.05.

## 4. Conclusions

The current study provides, for the first time, in vitro cholesterol transport data dependent on the differentiation stage of human primary trophoblast cells. Data reported in this study show significantly higher cholesterol transport capabilities in differentiated trophoblasts than undifferentiated trophoblasts and confirm the key role of LXR-sensitive transporter and receptor proteins in regulating transplacental cholesterol transport. The results in primary trophoblast cells demonstrate a predominantly apical cholesterol transport direction at term. These findings suggest that, at least at the end of pregnancy when terminal maturation of the fetus occurs, the ABCA1/apoA-1 pathway is rather involved in directing (excess) cholesterol back to the maternal circulation than in dispatching it to the far advanced developed fetus. Thus, the apoA-1/ABCA1 pathway seems to represent a protective and selective measure to prevent risks for the fetus arising from the potentially harmful antenatal exposure to excess cholesterol or cholesterol-derived hormones (e.g., steroid hormones).

## Figures and Tables

**Table 1:** Primer characteristics.

Gene	Accession Number	Primer Pairs (5’ end to 3’ end)	Product Length (bp)
*ABCA1*	XM_011518342.3	For: CCACATTTTTGCCTGGGACG Rev: AGCGATTCTCCCCAAACCTT	88
*ApoA-1*	NM_001318021.1	For: AGCGGCAGAGACTATGTG Rev: CTGTCCCAGTTGTCAAGG	83
*Endoglin*	NM_001114753.2	For: CAAGACCAGGAAGTCCATA Rev: CGTGTGCGAGTAGATGTA	174
*LDL-R*	XM_011528010.2	For: GACGTGGCGTGAACATCTG Rev: CTGGCAGGCAATGCTTTGG	111
*SR-BI*	NM_005505.4	For: CGGCTCGGAGAGCGACTAC Rev: GGGCTTATTCTCCATGATCACC	76
*SREBP-2*	XM_017028922.2	For: CCTTCCTCTGCCTCTCCTTT Rev: CACAAAGACGCTCAGGACAA	200
*YWHAZ*	XM_024447266.1	For: CCGTTACTTGGCTGAGGTTG Rev: AGTTAAGGGCCAGACCCAGT	143

Primers used for the quantitative RT-PCR measurements of *apoA-1* and *endoglin*, were designed using Beacon DesignerTM software. The remaining primers were taken from previous studies [14,39,40]. *ABCA1*: ATP-binding cassette transporter A1, *ApoA-1*: apolipoprotein A1, *LDL-R*: Low density lipoprotein receptor, *SR-B1*: scavenger receptor B 1, *SREBP-2*: sterol response element binding protein 2, and *YWHAZ*: Tyrosine 3-monooxygenase/tryptophan 5-monooxygenase activation protein, zeta polypeptide.

**Table 2:** Expression of selected cholesterol homeostasis genes and corresponding cholesterol uptake rates in CTB and STB.

	Steady State		LXR Agonist Stimulation	
	CTB	STB	CTB	STB
**mRNA Levels**				
*SR-B1* (×10^−3^)	13.4 ± 5.90	15.0 ± 5.95 ^A^	16.4 ± 4.90 ^X^	21.2 ± 5.98 ^B,Y^
*LDL-R* (×10^−3^)	14.8 ± 8.7 ^a,^*	15.8 ± 10.9 ^A^	67.1 ± 54.6 ^b,^*	44.5 ± 25.9 ^B^
*ABCA1* (×10^−3^)	13.7 ± 8.76 ^a^	21.0 ± 20.4 ^A^	132.8 ± 76.9 ^b^	117.8 ± 42.0 ^B^
*SREBP-2* (×10^−2^)	3.6 ± 2.2 ^a^	4.7 ± 4.4 ^A^	8.6 ± 5.5 ^b^	7.4 ± 3.2 ^B^
*ApoA-1* (×10^−5^)	6.9 ± 3.6	9.7 ± 12	6.6 ± 5	6.3± 2.7
**Cholesterol Uptake (CU)**				
% CU	13.5 ± 3.60	15.1 ± 3.98 ^A^	13.4 ± 3.75 ^X^	21.8 ± 3.49 ^B,Y^
**Correlation Coefficients**				
CU vs. SR-B1 mRNA	*r* = −0.3; *p* = 0.302	*r* = 0.5; *p* = 0.094	*r* = 0.2; *p* = 0.478	*r* = 0.2; *p* = 0.56
CU vs. LDL-R	*r* = −0.52; *p* = 0.078	*r* = −0.16; *p* = 0.60	*r* = 0.59; *p* = 0.04	*r* = −0.07; *p* = 0.80
CU vs. SREBP-2 mRNA	*r* = 0.7; *p* = 0.010	*r* = 0.6; *p* = 0.041	*r* = 0.8; *p* = 0.004	*r* = 0.2; *p* = 0.57

Data are mean ± SD of at least three independent experiments. ^a,b^: different small letters indicate significant (*p* < 0.05) treatment effect in CTB. ^A,B^: different capital letters indicate significant (*p* < 0.05) treatment effect in STB. ^X,Y^: different letters indicate significant (*p* < 0.05) differences between CTB and STB within each treatment regime (i.e., steady state and LXR agonist stimulation, respectively). T0901317 (0.1 µM) was used as LXR agonist. CTB: primary undifferentiated trophoblast cells, STB: differentiated trophoblast cells, *SR-B1*: scavenger receptor B 1, *LDL-R*: low density lipoprotein receptor, *ABCA1*: ATP-binding cassette transporter A1, *SREBP-2*: sterol response element binding protein 2; *apoA-1*: apolipoprotein A1. * *p* ≤ 0.1.
